# Effects of a 12-week Tai Chi program on postural stability and gait biomechanics in prefrail older adults: a randomized controlled study

**DOI:** 10.1186/s12984-026-02057-9

**Published:** 2026-06-24

**Authors:** Jiaxin Gao, Xueling Tu, Jianhao Chen, Sheng Huang, Zongxing Lu, Xiaofei Jia, Jingpeng Chang, Cai Jiang, Zhonghua Lin

**Affiliations:** 1 Rehabilitation Medicine Center, Fuzhou University Affiliated Provincial Hospital, Fuzhou, China; 2 Shengli Clinical Medical College of Fujian Medical University, Fuzhou, China; 3 Department of Rehabilitation Medicine, Quanzhou First Hospital Affiliated to Fujian Medical University, Quanzhou, China; 4 School of Mechanical Engineering and Automation, Fuzhou University, Fuzhou, China; 5 Department of Rehabilitation Medicine, General Hospital of Ningxia Medical University, Yinchuan, China; 6 Rehabilitation Medicine Center, People’s Hospital of Ningxia Hui Autonomous Region, Yinchuan, China; 7 Fujian Key Laboratory of Geriatrics, Fuzhou, China

**Keywords:** Tai Chi, Prefrail, Older adults, Gait, Postural stability

## Abstract

**Background:**

Prefrailty is a reversible transitional stage of frailty and is associated with impaired postural stability and increased fall risk. Tai Chi may improve balance-related performance in older adults, but its effects on postural stability and gait biomechanics in prefrail populations remain insufficiently characterized.

**Methods:**

In this single-blind randomized controlled trial, 68 prefrail older adults defined by the Fried Frailty Phenotype were allocated to a Tai Chi plus health education group or a health education control group. Sixty-four participants completed the study (mean age 71.1 years; 31 women; 33 men). The intervention group performed 24-form Tai Chi three times weekly for 12 weeks, while both groups received health education. Functional postural stability was assessed using the Timed Up-and-Go Test (TUGT), Berg Balance Scale (BBS), and Short Physical Performance Battery (SPPB). Static balance was evaluated using center-of-pressure (COP) measures, and gait biomechanics were assessed using three-dimensional gait analysis. Continuous outcomes were analyzed using baseline-adjusted ANCOVA, with complementary group × time interaction analyses. Exploratory change-score correlations were conducted within the intervention group, with full-sample sensitivity analyses adjusted for group assignment.

**Results:**

After 12 weeks, the intervention group showed better postural stability than controls, including shorter TUGT time (*P* < 0.001), higher BBS (FDR *q* < 0.001) and SPPB scores (FDR *q* = 0.002), lower COP path length and sway velocity under eyes-open conditions (both FDR *q* = 0.003), and lower COP path length, sway ellipse area, and sway velocity under eyes-closed conditions (FDR *q* = 0.014–0.018). Gait analysis showed significant improvements in gait speed (FDR *q* = 0.033), gait profile score (GPS; FDR *q* < 0.001), gait deviation index (GDI; FDR *q* < 0.001), and hip rotation deviation (FDR *q* = 0.007), whereas other joint-specific kinematic variables were only nominally significant. Group × time analyses supported changes in TUGT, BBS, SPPB, gait speed, GPS, and GDI. Exploratory correlation analyses showed that changes in postural stability were mainly associated with changes in hip kinematics, step length, and selected ankle-related features; however, these associations did not remain significant after FDR correction.

**Conclusion:**

A 12-week Tai Chi program improved functional performance and static postural control in prefrail older adults and was associated with favorable changes in gait speed and global gait-quality indices. Correlation findings should be interpreted as preliminary mechanistic clues. Larger studies with longer follow-up are needed.

*Trial registratio*n Chinese Clinical Trial Registry, ChiCTR2300073905, Registered on [2023-07-25]. https://www.chictr.org.cn.

## Introduction

With the acceleration of population ageing, frailty [1] has become a major public health issue affecting functional independence and quality of life in older adults [2]. Frailty is characterized by multisystem functional decline and reduced physiological reserve [3], which substantially increases vulnerability to stressors and the risk of hospitalization, disability, and other adverse health outcomes [4]. However, frailty should not be considered an irreversible end state. Its earlier stage, prefrailty [5], is more prevalent among community-dwelling older adults and is potentially reversible, making it a critical window for interventions aimed at preventing functional decline and adverse outcomes [6].

A Given that prefrailty represents an early and potentially reversible stage of frailty, identifying functional impairments that may contribute to further decline is of clear clinical relevance. Among these, impaired postural stability is commonly observed in older adults with frailty and prefrailty and is closely related to increased fall vulnerability [7]. Although prefrailty in the present study was defined using the Fried Frailty Phenotype, several characteristics captured by this framework, such as weakness, slowness, and low physical activity, may also be accompanied by reduced neuromuscular reserve and impaired balance control. Postural control depends on the integration of multisensory inputs, including visual, vestibular, and proprioceptive information [8], together with the coordinated regulation of lower-limb joints and trunk musculature [9]. Impairment of this system may lead to increased postural sway during quiet standing, reduced efficiency in dynamic postural transitions, and compromised gait stability [10, 11]. These changes may in turn increase fall risk and contribute to ongoing functional decline. Accordingly, interventions aimed at improving postural stability may be particularly relevant in prefrail populations.

Exercise interventions are widely regarded as effective strategies for preventing, delaying, or even reversing frailty, primarily by enhancing muscle strength and improving neuromuscular control and balance function to preserve functional independence in older adults [12]. Tai Chi [13], a traditional mind–body exercise characterized by gentle, low-intensity movements and a high safety profile, integrates elements of strength, balance, coordination, and attentional control, making it well suited to the training needs of prefrail older adults. Previous studies [14] and systematic reviews [15] have suggested that Tai Chi training may improve balance performance and postural control in older adults through increases in lower-limb muscle strength, joint range of motion, and neuromuscular coordination [16]. Nevertheless, evidence regarding the effects of Tai Chi on postural stability specifically in prefrail populations remains limited. Moreover, most existing studies have relied primarily on functional scales as outcome measures, which, while informative for overall functional improvement, provide limited insight into the underlying biomechanical characteristics and postural control strategies associated with these changes.

Beyond functional scales, balance platform assessment based on center-of-pressure (COP) measures can quantify sway-related parameters during quiet standing, including path length, sway velocity, and sway area [17], thereby providing a more objective and reproducible characterization of postural stability [11, 18]. Meanwhile, gait biomechanics offers complementary information from another key daily activity closely related to fall risk [19], thereby contributing to a more comprehensive understanding of motor function in prefrail older adults [5, 20]. Gait is not merely a process of forward progression, but a dynamic task requiring the continuous integration of postural control, body propulsion, and limb coordination. Its spatiotemporal parameters [21, 22] and lower-limb kinematic features [23–25], such as hip extension, ankle plantarflexion, and joint range of motion, not only reflect surface-level walking performance, such as gait speed and step length, but may also provide insight into propulsion capacity, support stability, and compensatory movement strategies [26]. In prefrail older adults, these measures may help detect early kinematic alterations that are not yet manifested as overt functional impairment, and may offer a more detailed biomechanical perspective for understanding intervention-related adaptations [27].

From a mechanistic perspective, functional postural performance, static sway control, and lower-limb coordination during walking are not independent domains, but share common foundations in sensory integration and neuromuscular control [28, 29]. Therefore, if an intervention improves postural stability, such changes may be reflected not only in improved functional scale performance and reduced static sway, but also in concurrent changes in selected gait spatiotemporal and kinematic characteristics. On this basis, exploring the associations between changes in postural stability and gait biomechanics may provide preliminary insight into intervention-related mechanisms. The combined use of functional scales, static balance platform assessment, and three-dimensional gait analysis may therefore offer a more comprehensive characterization of these changes and help interpret the potential functional adaptations from a gait biomechanical perspective.

Based on this rationale, the objectives of the present study were to: (1) examine the effects of a 12-week Tai Chi training program on functional and static postural stability in prefrail older adults; (2) systematically evaluate the impact of Tai Chi on spatiotemporal gait parameters and lower-limb kinematic characteristics during natural walking using three-dimensional gait analysis; and (3) explore whether post-intervention changes in postural stability were associated with changes in gait biomechanics, in order to provide preliminary insight into potential biomechanical correlates of the intervention.

## Methods

### Participants

Participants were screened according to the following eligibility criteria to ensure the inclusion of prefrail older adults who were able to safely participate in the intervention and complete all outcome assessments. Frailty status was determined using the Fried Frailty Phenotype [3, 30, 31], which consists of five components: unintentional weight loss, exhaustion, low physical activity, slowness, and weakness. Participants meeting one or two of these criteria were classified as prefrail.

The inclusion criteria were as follows: (1) Older adults aged 65–80 years; (2) Classified as prefrail according to the Fried Frailty Phenotype, with a score of 1–2; (3) Able to walk independently without assistive devices; (4) Berg Balance Scale (BBS) score > 40, used as a pragmatic safety-related eligibility criterion to ensure adequate balance and ambulatory capacity for safe participation in Tai Chi training and gait assessments [32–34]; (5) No major limb dysfunction that would prevent participation in exercise;(6) Willing to provide written informed consent and comply with all study procedures throughout the intervention period.

The exclusion criteria were as follows: (1) Severe cardiovascular, pulmonary, hepatic, renal, or other major systemic diseases; (2) Severe osteoarthritis or musculoskeletal disorders causing marked pain or preventing standardized gait testing; (3) Neurological disorders affecting gait, such as foot drop, hemiplegia, or Parkinson’s disease; (4) Major psychiatric illness or severe psychological disorders; (5) Visual, auditory, or cognitive impairment that would interfere with training participation or outcome assessment.

The withdrawal criteria were as follows: (1) Participation in other structured exercise or rehabilitation programs during the intervention period; (2) Failure to comply with the study protocol or refusal to complete outcome assessments; (3) Poor adherence to the intervention (Tai Chi attendance < 80%); (4) Accident, newly developed condition, or serious adverse event affecting walking ability or preventing continued participation.

### Sample size

The sample size was calculated based on the time required to complete the Timed Up-and-Go test (TUGT), which was used as the primary outcome indicator for improvement in postural stability control ability [35]. According to a preliminary pilot study, baseline TUGT performance was comparable between groups, while the post-intervention TUGT time was 10.17 ± 0.96 s in the intervention group and 11.08 ± 1.32 s in the control group. Using G*Power 3.1 software, the calculated effect size was 0.787. With the significance level set at α = 0.05 and statistical power at 0.80, the minimum required sample size was estimated to be 27 participants per group. Allowing for a 20% dropout rate, 34 participants were required in each group. Therefore, the total sample size for this study was 68 participants, with 34 participants allocated to the intervention group and 34 to the control group.

### Study design

This 12-week single-blind randomized trial was conducted between August 2023 and October 2024, with participant recruitment and intervention implemented at the Fuzhou University Affiliated Provincial Hospital and surrounding community settings. A simple randomization method was used to ensure the randomness and methodological rigor of participant allocation. First, 68 random numbers were generated using a computer and randomly assigned in a 1:1 ratio to group A (intervention group) and group B (control group), with 34 participants in each group. To ensure allocation concealment and strict implementation of the randomization procedure, the assignment results were sealed in sequentially numbered, opaque envelopes. Each envelope contained the treatment allocation for the corresponding participant and was ordered according to the randomization sequence. Upon enrollment, researchers opened the envelopes sequentially and assigned participants to the corresponding study group (intervention or control) based on the allocation indicated inside. The researcher responsible for managing the randomization process did not participate in participant recruitment, admission, or treatment, thereby ensuring the objectivity and independence of the allocation process. Owing to the distinctive and readily identifiable nature of the intervention (Tai Chi training), double blinding of participants and intervention providers was not feasible. Therefore, only outcome assessors and statistical analysts were blinded to group allocation to minimize potential bias during data collection and analysis.

Ethical approval for this study was obtained from the Hospital Ethics Committee prior to study initiation (Approval No. K2022-09-029). All participants and their family members were informed of the study objectives, and written informed consent was obtained. The trial was registered with the Chinese Clinical Trial Registry on July 25, 2023 (Registration No. ChiCTR2300073905).

### Interventions

#### Control group (health education)

Health education: Participants in the control group attended health education sessions only, conducted once every two weeks for 45 min each session, over a 12-week period, for a total of six sessions. During the study period, participants in the control group were instructed to maintain their usual daily routines and not to engage in any planned physical training activities, such as walking or brisk walking. The health education sessions were delivered by rehabilitation physicians and focused on education related to frailty syndrome in older adults, including its etiology, pathophysiological mechanisms, clinical manifestations, and strategies for prevention and management.

#### Intervention group (health education + Tai Chi)

Health education was identical to that provided to the control group. The Tai Chi program selected for this study was the simplified 24-form Tai Chi, which was developed by the General Administration of Sport of China based on Yang-style Tai Chi. The reference manual was ‘Twenty-Four Form Tai Chi”, published by People’s Sports Publishing House in 2007. The specific movements included: (1) Commencing Form, (2) Parting the Wild Horse’s Mane (left and right), (3) White Crane Spreads Its Wings, (4) Brush Knee and Twist Step (left and right), (5) Play the Lute, (6) Repulse Monkey (left and right), (7) Grasp the Bird’s Tail (left), (8) Grasp the Bird’s Tail (right), (9) Single Whip, (10) Wave Hands Like Clouds, (11) Single Whip, (12) High Pat on Horse, (13) Right Heel Kick, (14) Strike Opponent’s Ears with Both Fists, (15) Turn and Left Heel Kick, (16) Snake Creeps Down and Golden Rooster Stands on One Leg (left), (17) Snake Creeps Down and Golden Rooster Stands on One Leg (right), (18) Fair Lady Works at Shuttles (left and right), (19) Needle at Sea Bottom, (20) Flash the Arms, (21) Turn, Deflect, Parry, and Punch, (22) Apparent Close-Up, (23) Cross Hands, and (24) Closing Form.

Each training session consisted of three phases: a 10-min warm-up, 50 min of Tai Chi practice, and a 10-min cool-down. Training was conducted three times per week on nonconsecutive days for 12 weeks, resulting in a total of 36 sessions.

Tai Chi instruction was delivered by an experienced coach specializing in Tai Chi teaching to ensure the scientific rigor and standardization of the training content. The intervention was implemented in batches through centralized, fixed-site sessions, with participants in each batch trained as a single large group to maintain consistency and continuity of the program. Each session was supervised throughout by members of the research team, who promptly documented participants’ training status, including attendance, movement proficiency, and individual adaptation. To further enhance training quality, a national-level Tai Chi coach was invited weekly to provide technical guidance, with emphasis on movement standardization and key technical elements.

### Outcome measures

The selected outcomes were intended to capture complementary domains of postural stability and gait function. The Timed Up-and-Go Test (TUGT) was prespecified as the sole primary outcome. The Berg Balance Scale (BBS) and Short Physical Performance Battery (SPPB) were treated as key secondary clinical outcomes. Static postural control was assessed using center-of-pressure (COP)-based measures, and gait biomechanics were evaluated using three-dimensional gait analysis. Outcome assessments were conducted at baseline and after completion of the 12-week intervention. At each time point, all outcome measures were administered within a single assessment session, with sufficient rest intervals between tests to minimize fatigue-related effects.

#### Clinical postural stability outcomes

##### Timed up-and-go test (TUGT)

TUGT [36, 37] was used as the primary outcome to assess dynamic functional mobility and postural control. Participants were instructed to perform the test at a self-selected comfortable and safe pace: standing up from a chair, walking to a floor marker 3 m ahead, turning around, walking back to the chair, and sitting down. The turning direction was not standardized, and participants turned according to their natural preference. No physical assistance was provided during the test. One practice trial, which was not timed, was performed before a single timed trial. The total completion time of the timed trial was recorded and used for subsequent statistical analysis [38].

##### Berg balance scale (BBS)

The BBS [32] was used to evaluate overall clinical balance performance. It provides an overall assessment of static and dynamic balance in both sitting and standing positions. The BBS is widely used in clinical practice as a standardized measure of balance and demonstrates good reliability, validity, and sensitivity. The scale consists of 14 items, each item is scored on a 5-point scale ranging from 0 to 4, yielding a maximum total score of 56 points. The final score is the sum of all item scores, with lower scores indicating poorer balance function.

##### Short physical performance battery (SPPB)

The Short Physical Performance Battery (SPPB) [39] was used to assess lower-extremity physical performance. It includes tests of standing balance, gait speed, and repeated chair rise, with a total score ranging from 0 to 12. Higher scores indicate better physical performance.

#### Static postural control outcomes

Static balance ability under eyes-open and eyes-closed conditions was assessed using the Huber 360 balance evaluation and training system [40] (Huber 360 Neuromuscular Control Training and Evaluation System; DJO, USA). Participants stood upright on the force platform with both feet while completing a 50-s test under each visual condition, during which COP trajectory parameters were collected. The specific procedure was as follows: a positioning frame was mounted on the force platform for standardization; participants stood barefoot with the medial borders of both feet aligned with the edges of the positioning frame, arms hanging naturally at the sides, maintaining an upright posture with the gaze directed horizontally, and completed the test according to system prompts. The primary static balance outcome measures included: (1) mean sway velocity (mm/s), reflecting the temporal characteristics of COP displacement, with higher values indicating reduced postural control stability; (2) path length (mm), quantifying the cumulative displacement of the COP trajectory and representing the overall magnitude of postural sway, with higher values indicating decreased postural stability; and (3) sway ellipse area (mm²), describing the spatial distribution of the COP trajectory and reflecting the spatial variability of the postural control system. An increased ellipse area indicates diminished postural control ability, and this parameter has significant diagnostic value in clinical assessment and evaluation of intervention effects.

#### Gait biomechanics outcomes

##### Gait data acquisition

Gait data were collected using the SMART-DX 400 infrared motion capture system (BTS Bioengineering, Italy). The system comprised eight infrared cameras (200 Hz), two time-synchronized video cameras (BTS eVixta, 50 Hz), and a dedicated acquisition and processing workstation. Reflective markers were placed on anatomical landmarks according to the system protocol (Davis Heel model) [41, 42].

A total of 22 markers were used, including 18 skin-mounted markers and 4 strap-mounted cluster markers. Specifically, skin-mounted markers were placed on: the seventh cervical spinous process (C7) − 1, bilateral acromion processes − 2, bilateral anterior superior iliac spines (ASIS) − 2, the midpoint of the line connecting the bilateral posterior superior iliac spines (PSIS) − 1, bilateral greater trochanters − 2, bilateral lateral femoral epicondyles − 2, bilateral fibular heads − 2, bilateral lateral malleoli − 2, bilateral fifth metatarsophalangeal joints − 2, and bilateral heels − 2. Four strap-mounted markers were positioned at the midpoints of the greater trochanter–lateral femoral epicondyle segments (bilateral thighs) and the midpoints of the fibular head–lateral malleolus segments (bilateral shanks) using elastic straps. To reduce clothing-related artifacts, small openings were made at key pelvic landmarks (e.g., ASIS and greater trochanter) to allow direct skin attachment; markers were reinforced with medical-grade adhesive tape to minimize wobble and detachment. Marker placement was performed by two assessors who had completed standardized training and passed internal competency assessment. For consistency, each participant’s markers were placed by a single assessor. The two assessors conducted regular calibration sessions every two weeks with periodic cross-checking of landmark identification and placement procedures.

After all markers were placed, participants were instructed to walk back and forth along an approximately 6-meter-long path five times at their most comfortable and natural walking speed, in order to collect gait data representative of habitual walking. Trials were discarded if participants reported obvious discomfort, exhibited atypical gait patterns, or if marker detachment occurred during walking, resulting in incomplete marker trajectories unsuitable for subsequent analysis. At least three valid trials were required; if fewer than three valid trials were obtained, additional trials were collected to meet the minimum data-quality requirement. When more than three valid trials passed quality control, three trials were selected for subsequent analysis according to predefined criteria (clear marker trajectories, good signal quality, and a gait pattern closest to the participant’s usual walking). Gait outcome measures included spatiotemporal parameters and kinematic parameters.

##### Spatiotemporal gait parameters

The selected spatiotemporal parameters included stance time, swing time, stance phase, swing phase, double-support phase, walking speed, cadence, step width, stride length, and step length. These parameters were used to evaluate walking ability and walking efficiency during gait.

##### Kinematic parameters

Kinematic parameters included gait deviation indices and sagittal-plane joint kinematic variables. Gait Variable Scores (GVS), Gait Profile Score (GPS), and Gait Deviation Index (GDI) were calculated by the BTS SmartClinic software using the normative reference dataset embedded in the system under the Davis Heel model. This reference dataset represents gait patterns from healthy individuals without known gait pathology and was used to quantify the degree to which each participant’s gait pattern deviated from healthy reference gait patterns. Specifically, GVS quantifies the degree of gait deviation for a specific joint in a specific plane of motion, with lower values indicating that the joint movement pattern is closer to the healthy reference pattern. GPS [43] is an overall composite derived from individual GVS values and reflects the overall deviation of an individual’s gait pattern from the healthy reference data; lower GPS values indicate that the overall gait pattern is closer to the reference pattern. GDI [44, 45] is a global gait function index scaled with 100 as the normative reference. Scores greater than 100 indicate gait performance better than the typical reference level, whereas scores below 100 indicate greater gait deviation. In addition, sagittal-plane hip, knee, and ankle flexion-extension peak angles and range of motion (ROM) were extracted for analysis.

### Gait signal processing

Gait cycle processing was performed using BTS proprietary software SmartClinic. Marker trajectories were filtered using a dual-pass fourth-order low-pass Butterworth filter with a cutoff frequency of 6 Hz [46, 47]. Peak metrics were calculated based on the per-cycle, time-normalized waveform data exported from SmartClinic. Sagittal-plane hip, knee, and ankle peak angles and ROM were extracted from the entire time-normalized gait cycle, from 0% to 100%, rather than separately from the stance or swing phase. For each valid gait cycle, the maximum (MAX) and minimum (MIN) values were identified from the sagittal-plane joint angle waveform across the full gait cycle. Peak flexion was defined as the maximum value of the flexion-extension waveform, whereas peak extension was calculated as − MIN, so that positive peak extension values indicated extension beyond the neutral position, whereas negative values indicated that the joint remained in flexion at its most extended position. ROM was calculated as the difference between the maximum and minimum values of the original waveform.

### Statistical analysis

Data were analyzed using Python 3.12 and R 4.3. Normality of continuous variables was assessed using the Shapiro–Wilk test; data are presented as mean ± SD when approximately normal, or median (IQR) otherwise. Categorical variables are reported as n (%). Levene’s test was used to assess homogeneity of variance. Baseline between-group comparisons were performed using an independent-samples t test when normality and equal variances were satisfied, Welch’s t test when normality was acceptable but variances were unequal, and the Mann–Whitney U test when the normality assumption was violated. Categorical variables were compared using the chi-square (χ²) test. All continuous post-intervention outcomes were analyzed using analysis of covariance (ANCOVA), with the post-intervention value as the dependent variable, group as the fixed factor, and the corresponding baseline value as a covariate. The TUGT was prespecified as the sole primary outcome. The remaining outcomes were treated as secondary or exploratory outcomes, and false discovery rate (FDR) correction was applied within prespecified outcome families to account for multiple comparisons. As a complementary longitudinal analysis, linear mixed-effects models were additionally fitted for each continuous outcome, including fixed effects for group, time, and the group × time interaction, with participant as a random intercept. If model assumptions were notably violated, appropriate transformations or robust/non-parametric alternatives were considered. Correlation analyses were treated as exploratory and were based on change scores (Δ = post − pre). To reduce dimensionality while focusing on intervention-responsive measures, these analyses were restricted to representative variables showing nominal between-group differences in the ANCOVA analyses (uncorrected *P* < 0.05). The primary exploratory analysis used Spearman correlations within the intervention group, and a full-sample sensitivity analysis was additionally performed using partial Spearman correlations adjusted for group assignment. Benjamini–Hochberg FDR correction was applied within each predefined correlation family, and both nominal and FDR-adjusted results were reported. All tests were two-sided, with *P* < 0.05 considered statistically significant.

## Results

### Attrition

**Fig. 1:**
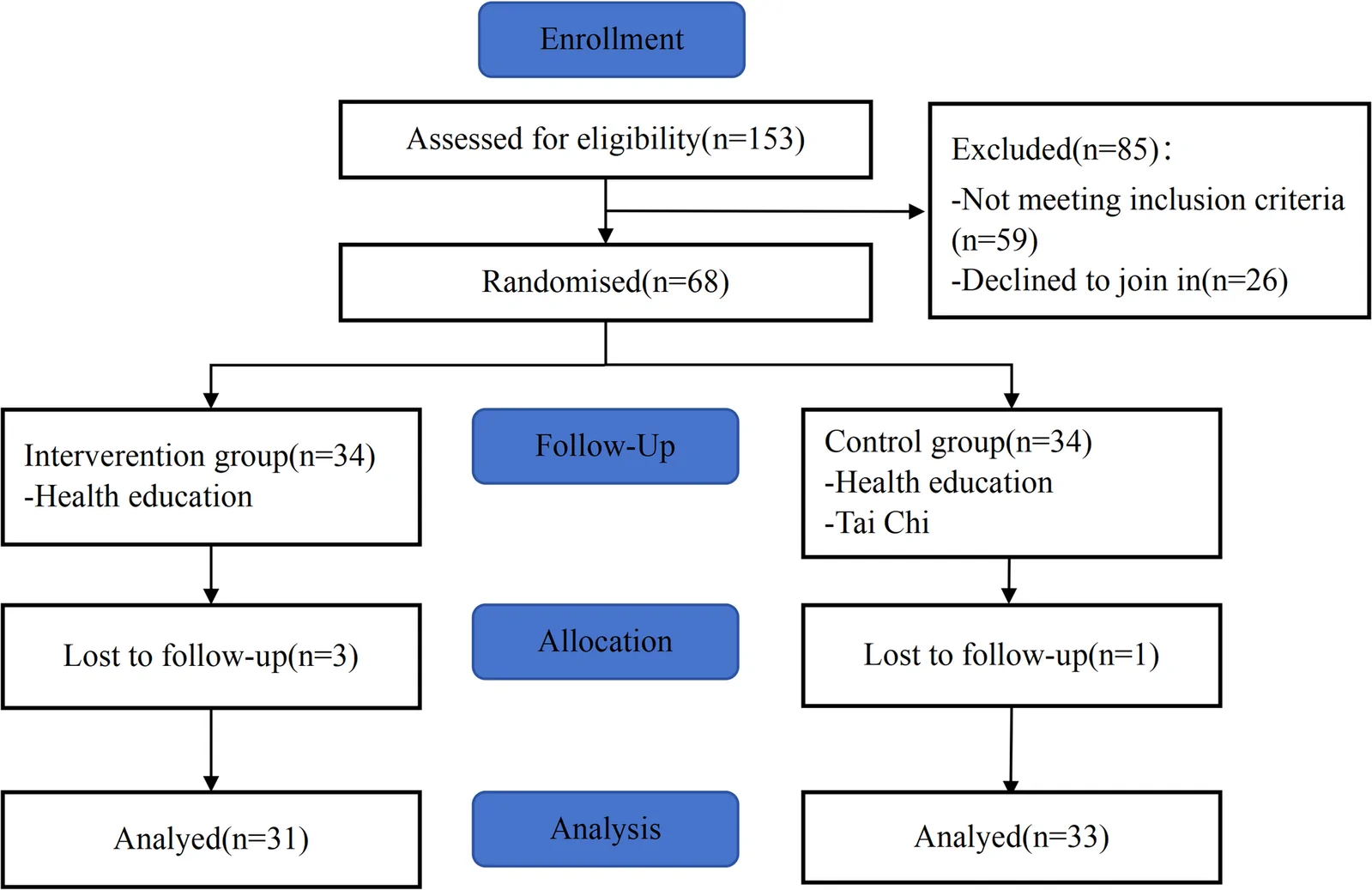
CONSORT flow diagram of the study

A total of 68 prefrail older adults were recruited from Fuzhou University Affiliated Provincial Hospital and surrounding communities. Following baseline assessments, participants were randomly allocated to two groups, with 34 participants in each group, and underwent a 12-week intervention. During the intervention period, 3 participants in the experimental group withdrew: one due to a fracture sustained in a bicycle-related fall, one due to withdrawal related to family caregiving responsibilities, and one who voluntarily withdrew because of personal family matters that prevented continued participation. In the control group, 1 participant withdrew due to overseas travel and was unable to complete the follow-up assessments. The flow of participants through screening, enrollment, allocation, follow-up, and analysis is illustrated in Fig. 1.

In total, 64 participants completed the post-intervention assessments, with 4 participants withdrawing from the study, yielding an overall attrition rate of 5.88%. Comparison of dropout rates between the two groups using Fisher’s exact test revealed no statistically significant difference (*P* > 0.05) (Table 1).

**Table 1:** Comparison of Dropout Rates between the Two Groups of Patients (n)

Group	Fall off				*P*
	Fracture	Voluntary withdrawal	Lost to follow-up	Total number	
IG	1	2	0	3	0.614
CG	0	0	1	1	

IG: intervention group; CG: control group

### Comparison of general demographic and clinical characteristics

No statistically significant differences were observed between the two groups in age, sex, height, weight, body mass index, exercise habits, baseline Fried Frailty Phenotype components, or attendance rate (all *P* > 0.05), indicating that the baseline characteristics and intervention adherence were comparable between groups (Table 2). All participants included in the final analysis met the prespecified adherence criterion of attending at least 80% of the planned supervised sessions.

**Table 2:** General demographic and clinical characteristics of the two groups (mean ± SD)

	IG (*n* = 31)	CG (*n* = 33)	t/Z/χ^2^/Fisher	*P*
Age(years)	70.19 ± 4.01	71.91 ± 4.4	-1.627	0.109
Sex(Male/Female)	15/16	18/15	0.243	0.622
Height(m)	1.62 ± 0.07	1.59 ± 0.08	1.554	0.125
Body mass(kg)	63.11 ± 8.64	61.90 ± 9.21	0.541	0.590
BMI(kg/m^2^)	24.01 ± 3.17	24.38 ± 3.23	-0.471	0.639
Regular exercise habit, n (%)	17 (54.8)	18 (54.5)	0.001	0.981
Exercise frequency, sessions/week	1.94 ± 2.16	2.06 ± 2.29	-0.114	0.915
**Exercise duration category**			0.964	0.810
No regular exercise, n (%)	14 (45.2)	15 (45.5)		
< 30 min/session, n (%)	2 (6.5)	4 (12.1)		
30–60 min/session, n (%)	12 (38.7)	10 (30.3)		
> 60 min/session, n (%)	3 (9.7)	3 (9.7)		
**Fried Frailty Phenotype components**				
Unintentional weight loss n (%)	2 (6.5%)	4 (12.1%)	0.605	0.673
Exhaustion n (%)	10 (32.3%)	11 (33.3%)	0.008	0.927
Low physical activity n (%)	11 (35.5%)	13 (39.4%)	0.104	0.747
Slowness n (%)	5 (16.1%)	6 (18.2%)	0.047	0.828
Weakness n (%)	8 (25.8%)	7 (21.2%)	0.188	0.665
One positive component n (%)	26 (83.9%)	25 (75.8%)	0.65	0.42
Two positive components n (%)	5 (16.1%)	8 (24.2%)	0.65	0.42
**Attendance rate (%)**	93.82% ± 5.50	92.93% ± 8.36	−0.310	0.757

IG: intervention group; CG: control group; BMI: body mass index

### Comparison of clinical postural stability outcomes

**Fig. 2:**
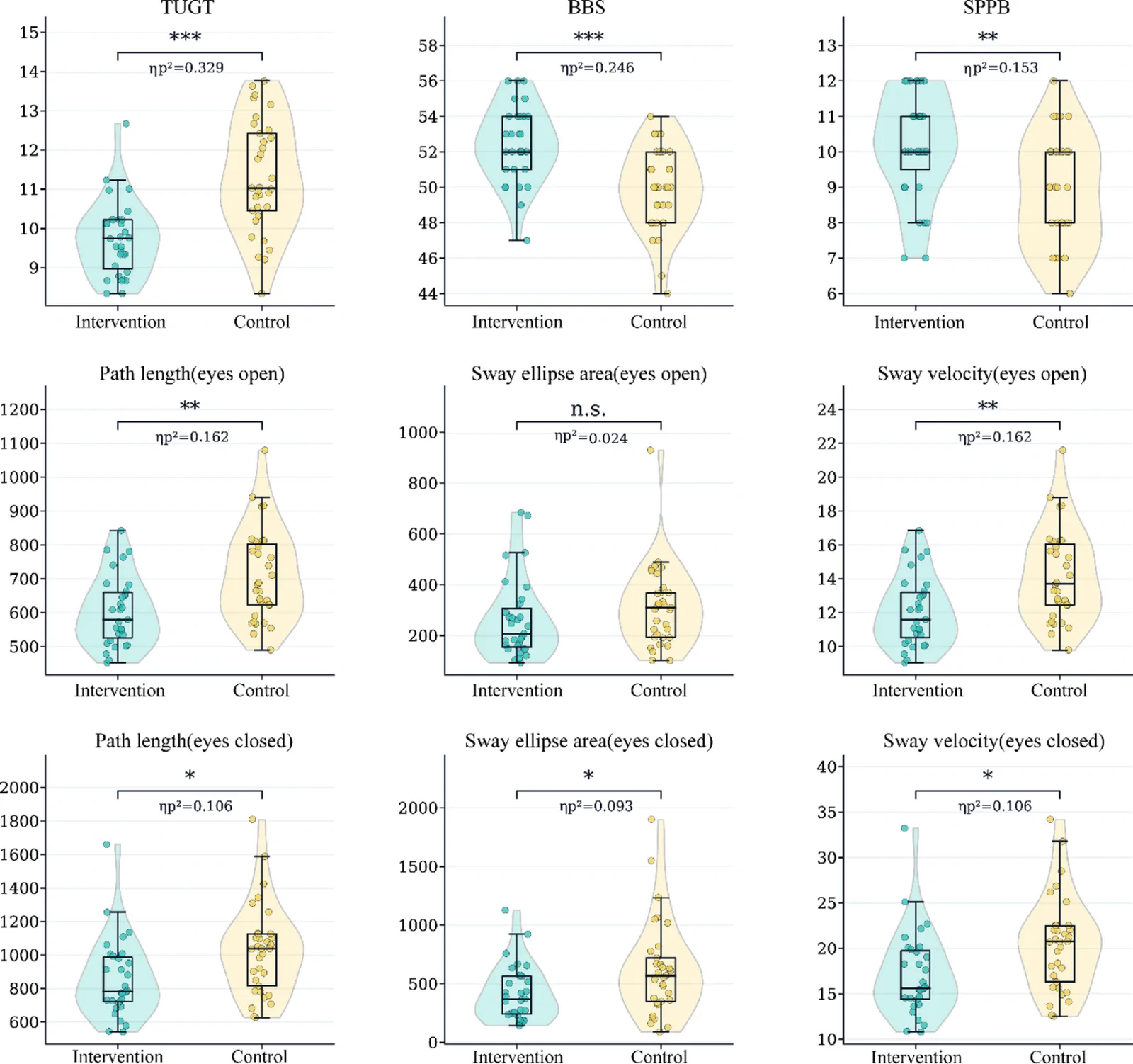
Raincloud plots comparing postural stability outcomes between groups after the intervention. Note: *: FDR *q* < 0.05; **: FDR *q* < 0.01; ***: FDR *q* < 0.001. (TUGT is reported based on the nominal P value)

After 12 weeks of intervention, baseline-adjusted ANCOVA showed that the intervention group had better post-intervention functional performance than the control group (Table 3; Fig. 2). TUGT, the prespecified primary outcome, was significantly lower in the intervention group (adjusted MD = − 1.63, 95% CI − 2.23 to − 1.04, *P* < 0.001). For the key secondary clinical outcomes, the intervention group also showed higher BBS scores (adjusted MD = 2.52, 95% CI 1.39 to 3.65, *P* < 0.001, FDR *q* < 0.001) and higher SPPB scores (adjusted MD = 1.27, 95% CI 0.50 to 2.03, *P* = 0.002, FDR *q* = 0.002), both of which remained significant after FDR correction within the clinical outcome family.

The complementary group × time interaction analysis was consistent with these findings (Table 4). Significant interactions were observed for TUGT (β = −1.79, 95% CI − 2.59 to − 0.98, *P* < 0.001), BBS (β = 2.27, 95% CI 0.55 to 3.98, *P* = 0.009, FDR *q* = 0.019), and SPPB (β = 0.97, 95% CI 0.01 to 1.93, *P* = 0.047, FDR *q* = 0.047), indicating greater improvement over time in the intervention group than in the control group.

**Table 3:** Changes in clinical postural stability outcomes in the two groups

Outcome	Intervention adjusted mean ± SE	Control adjusted mean ± SE	Adjusted between-group MD, 95% CI	*P* (Group)	FDR q	Partial η²
TUGT (s)	9.71 ± 0.21	11.34 ± 0.21	-1.63 (-2.25, -1.02)	< 0.001^***^		0.329
BBS (points)	52.43 ± 0.41	49.90 ± 0.39	2.52 (1.39, 3.65)	< 0.001^†^	< 0.001***	0.246
SPPB (points)	10.14 ± 0.27	8.87 ± 0.26	1.27 (0.50, 2.03)	0.002^†^	0.002**	0.153

SE: Standard Error; MD: mean difference (intervention - control)

^†^: Nominal *P* < 0.05

*: FDR *q* < 0.05

**: FDR *q* < 0.01

***: FDR *q* < 0.001 (TUGT is reported based on the raw P value)

**Table 4:** Group × time interaction results for clinical postural stability outcomes

Outcome	Group × time β (95% CI)	Interaction *p*	FDR q
TUGT (s)	1.79 (-2.59, -0.98)	< 0.001^†^	
BBS (points)	2.27 (0.55, 3.98)	0.009^†^	0.019*
SPPB (points)	0.09 (0.01, 1.93)	0.047^†^	0.047*

A negative interaction beta indicates a larger decrease over time in the intervention group relative to the control group

^†^: Nominal *P* < 0.05

*: FDR *q* < 0.05

### Comparison of static balance outcomes

For static balance outcomes (Table 5; Fig. 2), baseline-adjusted ANCOVA showed lower post-intervention COP path length and sway velocity under eyes-open conditions in the intervention group than in the control group (path length: adjusted MD = − 103.69, 95% CI − 164.02 to − 43.36, *P* = 0.001, FDR *q* = 0.003; sway velocity: adjusted MD = − 2.07, 95% CI − 3.28 to − 0.87, *P* = 0.001, FDR *q* = 0.003), whereas the between-group difference in eyes-open sway ellipse area was not significant (adjusted MD = − 48.95, 95% CI − 128.50 to 30.61, *P* = 0.223, FDR *q* = 0.223). Under eyes-closed conditions, the intervention group showed lower COP path length (adjusted MD = − 170.34, 95% CI − 297.24 to − 43.45, *P* = 0.009, FDR *q* = 0.014), smaller sway ellipse area (adjusted MD = − 206.12, 95% CI − 371.20 to − 41.05, *P* = 0.015, FDR *q* = 0.018), and lower sway velocity (adjusted MD = − 3.35, 95% CI − 5.84 to − 0.86, *P* = 0.009, FDR *q* = 0.014). Except for eyes-open sway ellipse area, all COP outcomes remained significant after FDR correction within the static balance family.

In contrast, the complementary group × time interaction analysis did not identify any COP outcome that remained significant after FDR correction (all FDR *q* ≥ 0.170, Table 6). Accordingly, the ANCOVA results support favorable post-intervention between-group differences in several COP measures, but the evidence for differential change over time in these static balance outcomes should be interpreted more cautiously.

**Table 5:** Changes in static balance outcomes in the two groups

Outcome	Intervention adjusted mean ± SE	Control adjusted mean ± SE	Adjusted between-group MD, 95% CI	*P* (Group)	FDR q	Partial η²
Path length (eyes open) (mm)	604.80 ± 21.64	708.49 ± 20.97	-103.69 (-164.02, -43.36)	0.001^†^	0.003**	0.162
Sway ellipse area (eyes open) (mm^2^)	263.59 ± 28.57	312.54 ± 27.69	-48.95 (-128.50, 30.61)	0.223	0.223	0.024
Sway velocity (eyes open) (mm/s)	12.10 ± 0.43	14.17 ± 0.42	-2.07 (-3.28, -0.87)	0.001^†^	0.003**	0.162
Path length (eyes closed) (mm)	853.97 ± 45.57	1024.31 ± 44.16	-170.34 (-297.24, -43.45)	0.009^†^	0.014*	0.106
Sway ellipse area (eyes closed) (mm^2^)	415.93 ± 59.21	622.05 ± 57.39	-206.12 (-371.20, -41.05)	0.015^†^	0.018*	0.093
Sway velocity (eyes closed) (mm/s)	17.08 ± 0.89	20.43 ± 0.87	-3.35 (-5.84, -0.86)	0.009^†^	0.014*	0.106

SE: Standard Error; MD: mean difference (intervention - control); ^†^: Nominal *P* < 0.05; *: FDR *q* < 0.05; **: FDR *q* < 0.01

**Table 6:** Group × time interaction results for static balance outcomes

Outcome	Group × time β (95% CI)	Interaction *P*	FDR q
Path length (eyes open) (mm)	-82.37 (-177.92, L813.19)	0.091	0.170
Sway ellipse area (eyes open) (mm^2^)	-54.27 (-165.33, 56.79)	0.338	0.338
Sway velocity (eyes open) (mm/s)	-1.65 (-3.56, 0.26)	0.091	0.170
Path length (eyes closed) (mm)	-161.72 (-359.92, 36.49)	0.110	0.170
Sway ellipse area (eyes closed) (mm^2^)	-148.65 (-405.47, 108.17)	0.257	0.308
Sway velocity (eyes closed) (mm/s)	-3.18 (-7.11, 0.76)	0.114	0.170

A negative interaction beta indicates a larger decrease over time in the intervention group relative to the control group

### Comparison of gait biomechanics outcomes

**Fig. 3:**
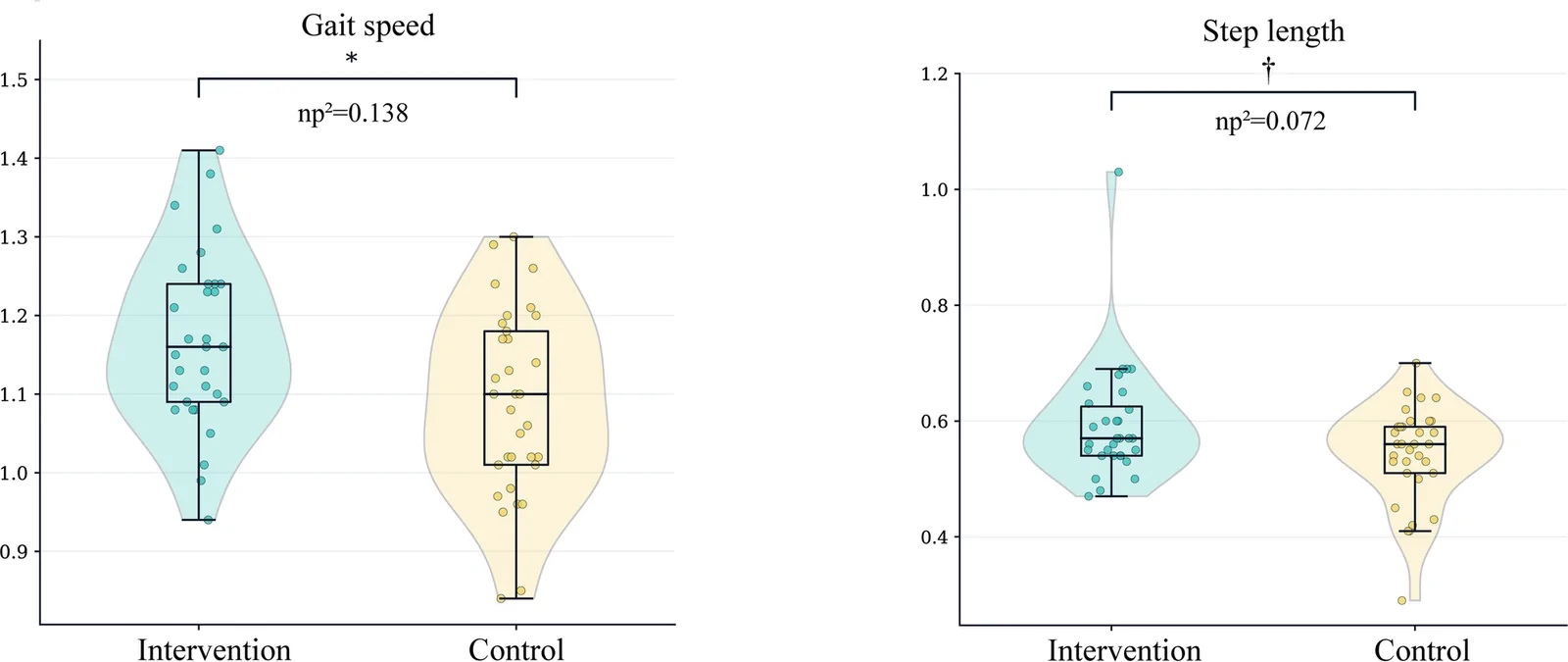
Raincloud plots comparing spatiotemporal gait parameters between groups after the intervention. Note: *: FDR *q* < 0.05; †: nominal *P* < 0.05 only

After adjusting for baseline values using ANCOVA, the intervention group showed overall better post-intervention gait performance than the control group, although only a subset of outcomes remained significant after FDR correction within each prespecified family. For spatiotemporal parameters (Tables 7; Fig. 3), gait speed (adjusted MD = 0.09, 95% CI = 0.03 to 0.14, *P* = 0.003, FDR *q* = 0.033, partial ηp² = 0.138) was significantly improved in the intervention group compared with the control group. Step length (adjusted MD = 0.05, 95% CI = 0.00 to 0.10, *P* = 0.034, partial ηp² = 0.072) showed a nominal between-group difference, but this did not remain significant after FDR correction.

**Fig. 4:**
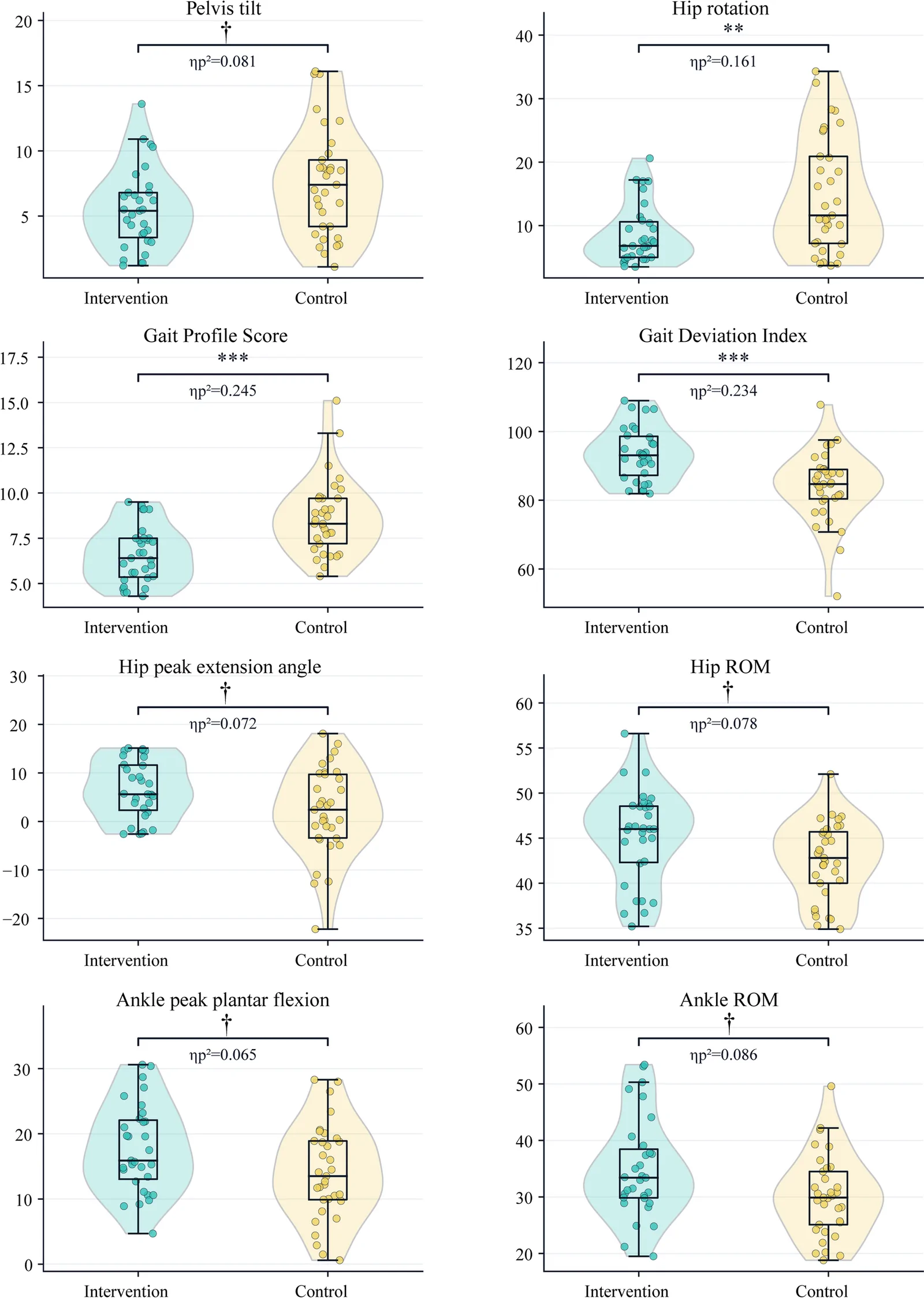
Raincloud plots comparing gait kinematic parameters between groups after the intervention. Note: *: FDR *q* < 0.05; **: FDR *q* < 0.01; ***: FDR *q* < 0.001; †: nominal *P* < 0.05 only; For ankle kinematics variable, positive values indicate that the joint moved beyond the neutral position in the specified direction, whereas negative values indicate that the joint remained in the opposite direction at its closest point to that direction

For kinematic parameters (Table 9; Fig. 4), the intervention group demonstrated a significantly lower GPS (adjusted MD = − 2.06, 95% CI = − 2.99 to − 1.13, *P* < 0.001, FDR *q* < 0.001, partial ηp² = 0.245) and a significantly higher GDI (adjusted MD = 9.67, 95% CI = 5.19 to 14.15, *P* < 0.001, FDR *q* < 0.001, partial ηp² = 0.234), indicating a marked improvement in overall gait quality. The GVS findings were partly consistent, showing that Hip rotation (adjusted MD = − 6.29, 95% CI = − 9.96 to − 2.63, *P* = 0.001, FDR *q* = 0.007, partial ηp² = 0.161) was significantly less abnormal in the intervention group than in the control group, whereas the between-group difference in Pelvis tilt (adjusted MD = − 2.16, 95% CI = − 4.02 to − 0.30, *P* = 0.023, partial ηp² = 0.081) did not remain significant after FDR correction. Regarding joint kinematics (Table 9; Fig. 4), the intervention group showed nominal advantages in Hip peak extension angle (adjusted MD = 4.25, 95% CI = 0.35 to 8.15, *P* = 0.033, partial ηp² = 0.072), Hip ROM (adjusted MD = 2.71, 95% CI = 0.32 to 5.09, *P* = 0.027, partial ηp² = 0.078), Ankle peak plantar flexion (adjusted MD = 3.66, 95% CI = 0.12 to 7.20, *P* = 0.043, partial ηp² = 0.065), and Ankle ROM (adjusted MD = 4.86, 95% CI = 0.68 to 9.03, *P* = 0.019, partial ηp² = 0.086), but none of these associations remained significant after FDR correction. No significant between-group differences were observed for the remaining outcomes.

The complementary group × time interaction analysis showed a generally similar pattern (Tables 8 and 10), with significant interactions retained after FDR correction for gait speed, GPS, and GDI, whereas the interaction for Hip rotation was nominally significant but did not remain significant after FDR adjustment.

**Table 7:** Comparison of spatiotemporal gait parameters

Outcome	Intervention adjusted mean ± SE	Control adjusted mean ± SE	Adjusted MD, 95% CI	*P* (Group)	FDR q	Partial η²
**Temporal parameters**						
Stride time (s)	1.09 ± 0.02	1.09 ± 0.02	-0.00 (-0.05, 0.05)	0.980	0.980	0
Stance time (s)	0.68 ± 0.01	0.68 ± 0.01	-0.00 (-0.04, 0.04)	0.954	0.980	0
Swing time (s)	0.41 ± 0.01	0.40 ± 0.01	0.01 (-0.01, 0.03)	0.477	0.817	0.008
Stance phase (%)	62.29 ± 0.95	62.72 ± 0.92	-0.42 (-3.09, 2.24)	0.753	0.980	0.002
Swing phase (%)	37.74 ± 0.52	37.08 ± 0.51	0.67 (-0.80, 2.13)	0.366	0.817	0.013
Single support phase (%)	38.45 ± 0.42	38.37 ± 0.41	0.08 (-1.09, 1.26)	0.89	0.980	0
Double support phase (%)	11.76 ± 0.41	12.18 ± 0.40	-0.42 (-1.57, 0.73)	0.467	0.817	0.009
Gait speed (m/s)	1.17 ± 0.02	1.08 ± 0.02	0.09 (0.03, 0.14)	0.003^†^	0.033*	0.138
Cadence (steps/min)	111.36 ± 1.73	112.03 ± 1.68	-0.67 (-5.51, 4.18)	0.784	0.980	0.001
**Spatial parameters**						
Stride length (m)	1.18 ± 0.03	1.10 ± 0.03	0.08 (-0.00, 0.15)	0.051	0.203	0.061
Step length (m)	0.59 ± 0.02	0.54 ± 0.02	0.05 (0.00, 0.10)	0.034^†^	0.203	0.072
Step width (m)	0.09 ± 0.01	0.10 ± 0.01	-0.01 (-0.03, 0.01)	0.289	0.817	0.021

SE: Standard Error; MD: mean difference (intervention - control); ^†^: Nominal *P* < 0.05; *: FDR *q* < 0.05

**Table 8:** Group × time interaction results for spatiotemporal gait parameters

Outcome	Group × time β (95% CI)	Interaction *P*	FDR q
**Temporal parameters**			
Stride time (s)	-0.03 (-0.10, 0.04)	0.384	0.651
Stance time (s)	-0.04 (-0.10, 0.02)	0.231	0.651
Swing time (s)	-0.01 (-0.04, 0.02)	0.542	0.651
Stance phase (%)	-1.80 (-5.15, 1.55)	0.293	0.651
Swing phase (%)	0.04 (-1.93, 2.02)	0.965	0.965
Single support phase (%)	0.61 (-1.27, 2.49)	0.523	0.651
Double support phase (%)	-0.90 (-2.70, 0.90)	0.329	0.651
Gait speed (m/s)	0.11 (0.04, 0.19)	0.003^†^	0.034*
Cadence (steps/min)	1.50 (-4.94, 7.94)	0.648	0.706
**Spatial parameters**			
Stride length (m)	0.12 (0.01, 0.23)	0.035^†^	0.212
Step length (m)	0.06 (-0.004, 0.12)	0.068	0.274
Step width (m)	-0.01 (-0.04, 0.02)	0.499	0.651

A negative interaction beta indicates a larger decrease over time in the intervention group relative to the control group. ^†^: Nominal *P* < 0.05; *: FDR *q* < 0.05

**Table 9:** Comparison of gait kinematic parameters

Outcome	Intervention adjusted mean ± SE	Control adjusted mean ± SE	Adjusted MD (95% CI)	*P* (Group)	FDR q	Partial η²
**Gait Variable Scores**						
Pelvis obliquity (deg)	2.05 ± 0.14	2.18 ± 0.17	-0.12 (-0.57, 0.32)	0.563	0.663	0.006
Pelvis tilt (deg)	5.46 ± 0.66	7.62 ± 0.64	-2.16 (-4.02, -0.30)	0.023^†^	0.090	0.081
Pelvis rotation (deg)	3.26 ± 0.21	3.64 ± 0.25	-0.38 (-1.03, 0.26)	0.253	0.421	0.021
Hip ab-adduction (deg)	4.13 ± 0.30	4.09 ± 0.39	0.04 (-0.94, 1.02)	0.931	0.933	0
Hip flex-extension (deg)	6.65 ± 0.43	8.42 ± 0.77	-1.76 (-3.51, -0.02)	0.052	0.115	0.061
Hip rotation (deg)	8.62 ± 0.84	14.91 ± 1.64	-6.29 (-9.96, -2.63)	0.001^†^	0.007**	0.161
Knee flex-extension (deg)	8.41 ± 0.54	9.77 ± 0.89	-1.36 (-3.44, 0.72)	0.194	0.353	0.027
Ankle dorsi-plantarflex (deg)	6.83 ± 0.58	7.01 ± 0.64	-0.18 (-1.90, 1.54)	0.832	0.903	0.001
Foot progression (deg)	7.80 ± 0.73	9.62 ± 0.71	-1.82 (-3.85, 0.22)	0.079	0.158	0.05
**Gait Profile Score (deg)**	6.58 ± 0.29	8.64 ± 0.36	-2.06 (-2.99, -1.13)	< 0.001^†^	< 0.001***	0.245
**Gait Deviation Index (score)**	93.58 ± 1.61	83.91 ± 1.56	9.67 (5.19, 14.15)	< 0.001^†^	< 0.001***	0.234
**Hip**,** Knee and Ankle kinematics in the sagittal plane**						
Hip peak flexion angle (deg)	38.73 ± 1.19	40.32 ± 1.16	-1.59 (-4.91, 1.74)	0.344	0.492	0.015
Hip peak extension angle (deg)	6.46 ± 1.40	2.22 ± 1.35	4.25 (0.34, 8.15)	0.033^†^	0.095	0.072
Hip ROM (deg)	45.22 ± 0.86	42.51 ± 0.83	2.71 (0.32, 5.09)	0.027^†^	0.090	0.078
Knee peak flexion angle (deg)	60.33 ± 1.51	58.98 ± 1.46	1.35 (-2.86, 5.56)	0.523	0.654	0.007
Knee peak extension angle (deg)	-2.89 ± 1.61	-3.29 ± 1.56	0.40 (-4.10, 4.91)	0.858	0.903	0.001
Knee ROM (deg)	57.33 ± 1.09	55.80 ± 1.06	1.53 (-1.52, 4.58)	0.320	0.492	0.016
Ankle peak dorsiflexion (deg)	17.26 ± 1.14	15.97 ± 1.10	1.28 (-1.88, 4.45)	0.420	0.560	0.011
Ankle peak plantar flexion (deg)	17.78 ± 1.27	14.12 ± 1.23	3.66 (0.12, 7.20)	0.043^†^	0.108	0.065
Ankle ROM (deg)	34.99 ± 1.64	30.13 ± 1.28	4.86 (0.68, 9.03)	0.019^†^	0.090	0.086

SE: Standard Error; MD: Mean difference (intervention - control); ^†^: Nominal *P* < 0.05 only; *: FDR *q* < 0.05; **: FDR *q* < 0.01; ***: FDR *q* < 0.001; ROM: Range of motion; For ankle kinematics variable, positive values indicate that the joint moved beyond the neutral position in the specified direction, whereas negative values indicate that the joint remained in the opposite direction at its closest point to that direction

**Table 10:** Group × time interaction results for gait kinematic parameters

Outcome	Group × time β (95% CI)	Interaction *P*	FDR q
**Gait Variable Scores (deg)**			
Pelvis obliquity (deg)	0.1 (-0.62, 0.82)	0.786	0.789
Pelvis tilt (deg)	-3.64(-6.36,-0.91)	0.009^†^	0.059
Pelvis rotation (deg)	-0.78(-1.73,0.17)	0.109	0.245
Hip ab-adduction (deg)	-0.2(-1.55,1.15)	0.771	0.789
Hip flex-extension (deg)	-2.09(-4.66,0.48)	0.110	0.245
Hip rotation (deg)	-5.77(-11.28,-0.26)	0.040^†^	0.161
Knee flex-extension (deg)	-1.18(-3.6,1.24)	0.338	0.483
Ankle dorsi-plantarflex (deg)	-0.35(-2.52,1.82)	0.754	0.789
Foot progression (deg)	-1.91(-4.91,1.09)	0.212	0.424
**Gait Profile Score (deg)**	-2.21(-3.56,-0.86)	0.001^†^	0.014*
**Gait Deviation Index (score)**	10.44(4.15,16.73)	0.001^†^	0.014*
**Hip**,** Knee and Ankle kinematics in the sagittal plane**			
Hip peak flexion angle (deg)	-2.49(-7.07,2.1)	0.288	0.443
Hip peak extension angle (deg)	6.24(0.87,11.62)	0.023^†^	0.114
Hip ROM (deg)	3.76(-0.17,7.69)	0.061	0.203
Knee peak flexion angle (deg)	3.21(-2.45,8.87)	0.266	0.443
Knee peak extension angle (deg)	-2.63(-8.58,3.32)	0.386	0.483
Knee ROM (deg)	0.58(-3.67,4.83)	0.789	0.789
Ankle peak dorsiflexion (deg)	1.79(-2.25,5.84)	0.384	0.483
Ankle peak plantar flexion (deg)	2.66(-1.93,7.26)	0.256	0.443
Ankle ROM (deg)	4.46(-0.66,9.58)	0.088	0.245

A negative interaction beta indicates a larger decrease over time in the intervention group relative to the control group. ^†^: Nominal *P* < 0.05; *: FDR *q* < 0.05

### Correlation analysis between changes in postural stability and gait biomechanics

**Fig. 5:**
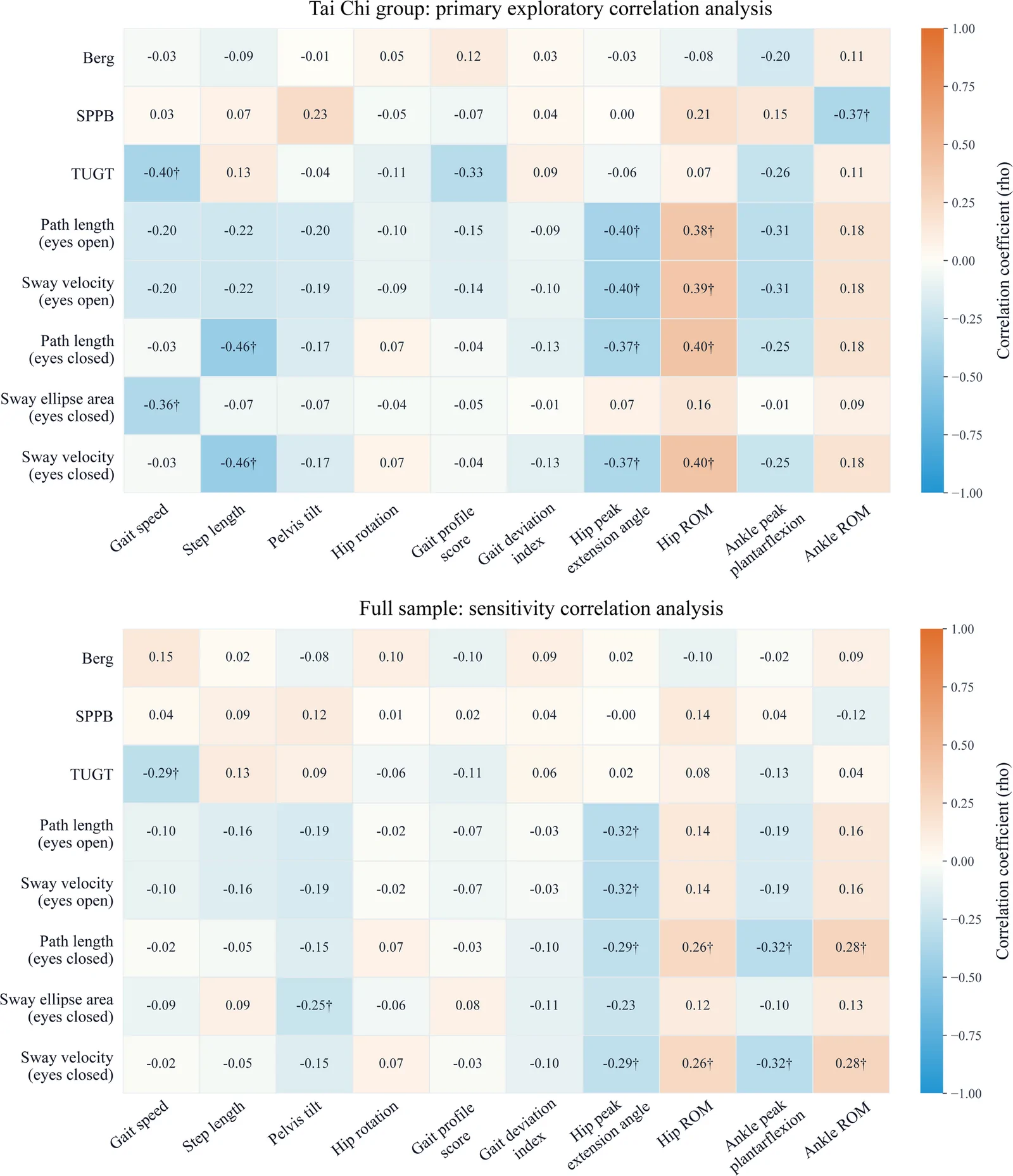
Correlation heatmap between postural stability outcome measures and gait parameters. Note: Upper panel: Tai Chi group, primary exploratory correlation analysis; lower panel: full sample, sensitivity correlation analysis. Cell values represent correlation coefficients. Significance markers are defined as follows: **: FDR *q* < 0.01; *: FDR *q* < 0.05; †: nominal *P* < 0.05 only

To further explore potential biomechanical correlates of post-intervention improvements in postural stability, exploratory correlation analyses were conducted using change scores (Δ = post − pre). To reduce dimensionality while focusing on intervention-responsive outcomes, the analyses were restricted to representative variables that showed nominal between-group differences in the ANCOVA analyses. In the primary exploratory analysis within the intervention group (Fig. 5, Upper panel), for Clinical Postural Stability Outcomes, ΔTUGT was negatively correlated with Δgait speed (Spearman’s ρ = −0.396, *P* = 0.027), and ΔSPPB was negatively correlated with Δankle ROM (ρ = −0.372, *P* = 0.040). For static balance under eyes-open conditions, both Δpath length and Δsway velocity were negatively correlated with Δhip peak extension angle (ρ = −0.400, *P* = 0.026; and ρ = −0.398, *P* = 0.027, respectively), and positively correlated with Δhip ROM (ρ = 0.381, *P* = 0.034; and ρ = 0.389, *P* = 0.030, respectively). For static balance under eyes-closed conditions, both Δpath length and Δsway velocity were negatively correlated with Δstep length (ρ = −0.464, *P* = 0.009 for both), positively correlated with Δhip ROM (ρ = 0.404, *P* = 0.024 for both), and negatively correlated with Δhip peak extension angle (ρ = −0.368, *P* = 0.041 for both). In addition, Δsway ellipse area under eyes-closed conditions was negatively correlated with Δgait speed (ρ = −0.355, *P* = 0.050). However, after Benjamini–Hochberg FDR correction across the full predefined correlation family, none of these associations remained significant.

In addition, we further performed a full-sample sensitivity analysis adjusted for group assignment using partial Spearman correlations. A broadly similar pattern of nominal associations was observed (Fig. 5, lower panel). For Clinical Postural Stability Outcomes, ΔTUGT was negatively associated with Δgait speed (partial ρ = −0.292, *P* = 0.019). For static balance under eyes-open conditions, both Δpath length and Δsway velocity were negatively associated with Δhip peak extension angle (partial ρ = −0.320, *P* = 0.010; and ρ = −0.319, *P* = 0.010, respectively). For static balance under eyes-closed conditions, both Δpath length and Δsway velocity were negatively associated with Δankle peak plantar flexion (ρ = −0.318, *P* = 0.010 for both), negatively associated with Δhip peak extension angle (ρ = −0.286, *P* = 0.022 for both), positively associated with Δankle ROM (ρ = 0.277, *P* = 0.027 for both), and positively associated with Δhip ROM (ρ = 0.257, *P* = 0.041 for both). In addition, Δsway ellipse area under eyes-closed conditions was negatively associated with Δpelvis tilt (ρ = −0.249, *P* = 0.048). Nevertheless, none of the full-sample associations remained significant after FDR correction. Accordingly, these findings are better interpreted as exploratory signals rather than robust evidence of stable biomechanical associations.

### Adverse events

During the trial period, participants in both groups were monitored for safety-related events through researcher observation and participant reporting during intervention and assessment sessions. The research team recorded events such as falls, injuries, marked pain, and any events requiring medical evaluation or treatment. No serious adverse events or clinically significant adverse events attributable to the Tai Chi intervention were observed.

## Discussion

### Key findings

This study demonstrates that a 12-week Tai Chi training program confers multidimensional balance benefits in prefrail older adults. At baseline, all participants met the Fried Frailty Phenotype criterion for prefrailty, defined as the presence of one or two positive Fried components. The distribution of positive Fried components was comparable between the intervention and control groups, supporting the baseline comparability of frailty phenotype characteristics. After the 12-week intervention, functional assessments showed that the intervention group had a significantly shorter TUGT completion time and significantly higher BBS and SPPB scores, and these differences remained significant after FDR correction, suggesting improvements in overall mobility and balance function. Static postural control assessment further showed significant reductions in COP sway-related parameters during static standing tasks in the intervention group, including path length, sway velocity, and sway area, with these differences also remaining significant after FDR correction, suggesting improved postural control under static conditions. Three-dimensional gait analysis further indicated that Tai Chi training was associated with improved gait performance. After FDR correction within prespecified outcome families, the intervention group showed significantly greater gait speed, lower GPS, higher GDI, and reduced hip rotation deviation compared with the control group, suggesting improvements in both walking efficiency and overall gait quality. Several additional kinematic variables, including step length, pelvic tilt deviation, hip peak extension angle, hip ROM, ankle peak plantar flexion, and ankle ROM, showed only nominal between-group differences and did not remain significant after FDR correction; therefore, these findings should be interpreted as exploratory. Collectively, converging evidence from functional scales, objective static postural control assessment, and three-dimensional gait analysis supports the beneficial effects of Tai Chi on postural stability and gait performance in prefrail older adults.

### Improvement in postural stability: from functional balance to static sway control

TUGT, BBS, and SPPB primarily reflect integrated functional performance during daily activities, including sit-to-stand transitions, turning, walking, and postural adjustments. Improvements in these measures suggest that Tai Chi training may enhance functional mobility and balance-related performance in prefrail older adults [35, 48, 49]. In the present study, these clinical outcomes showed a relatively consistent pattern, with favorable between-group differences after baseline adjustment and corresponding support from the complementary group × time interaction analysis. When interpreting these improvements in functional outcomes, their clinical relevance should also be considered. In the present study, the adjusted between-group difference in SPPB was 1.27 points, exceeding the commonly cited threshold of 1.0 point for substantial meaningful change in older adults [50]. By contrast, minimal Clinically Important Difference (MCID) estimates for TUGT and BBS are more population- and condition-specific, and validated MCID values for prefrail older adults have not been firmly established [51, 52]. Overall, although these findings should be interpreted cautiously, the observed improvements in TUGT and BBS appear clinically relevant in the broader context of improved functional mobility and balance performance.

For static balance, the intervention group showed lower COP sway parameters under both eyes-open and eyes-closed conditions in the ANCOVA analysis, including reductions in path length and sway velocity in both conditions and a lower sway area under eyes-closed conditions. These findings suggest improved static postural control, reflected by a smaller range of postural sway, lower sway velocity, and a more stable overall postural control pattern. However, widely accepted MCID thresholds for COP-based postural sway parameters, including COP path length, sway velocity, and sway area, have not been established for prefrail older adults [51]. Therefore, the clinical relevance of these COP findings was interpreted primarily based on the consistent reduction in sway-related parameters and their convergence with improvements in functional balance outcomes. Previous studies have reported that Tai Chi, characterized by slow weight shifting, single-leg support, and multidirectional stepping, may enhance postural regulation capacity and static balance performance [53]. The improvements observed under eyes-closed conditions further suggest that Tai Chi may improve balance not only through visually guided control [54] but also by promoting the integration and re-weighting of proprioceptive and vestibular information [55, 56]. Nevertheless, after correction for multiple comparisons, the group × time interaction analysis did not provide equally robust support for COP-based outcomes. Therefore, although the present findings suggest potential benefits of Tai Chi for static postural control under both standard and sensory-restricted conditions, the COP-related results should be interpreted with caution and confirmed in larger studies of prefrail populations.

### Changes in gait biomechanics

Three-dimensional gait analysis indicated that the intervention group showed more favorable post-intervention gait biomechanics than the control group, although the more consistent findings were concentrated in only a subset of outcomes after correction for multiple comparisons. In the spatiotemporal domain, only gait speed remained significant after FDR correction, whereas the between-group difference in step length was no longer significant. In the kinematic and gait quality domain, the intervention group showed a lower GPS, a higher GDI, and a lower hip rotation deviation, suggesting less deviation in overall gait pattern and less rotational abnormality [57]. By contrast, several joint-specific kinematic variables, including pelvic tilt, hip extension, hip range of motion, ankle plantarflexion, and ankle range of motion, showed nominal between-group differences in the expected direction, but these associations did not remain significant after FDR correction. This pattern may be related to the relatively small sample size of the present study, as well as the greater inter-individual variability typically seen in joint-specific kinematic measures, both of which may reduce statistical power after adjustment for multiple comparisons. Therefore, these findings should not be interpreted as conclusive evidence of broad kinematic adaptation.

Notably, GPS and GDI have rarely been used in previous Tai Chi studies. Most prior studies have focused on gait speed, step length, cadence, balance function, or selected joint kinematic parameters, whereas global gait-quality indices based on three-dimensional gait analysis have been less frequently applied [53]. GPS and GDI can summarize complex three-dimensional kinematic data and quantify the overall deviation of an individual’s gait pattern from healthy reference gait [43–60]. Therefore, including these two indices enabled a more integrative evaluation of Tai Chi-related gait changes at the whole-kinematic-pattern level and represents an important feature that distinguishes the present study from most previous studies [43]. In the present study, the adjusted between-group difference was − 2.06° for GPS and + 9.67 points for GDI, suggesting that Tai Chi training may not only increase gait speed but also improve overall gait kinematic quality. Previous rehabilitation studies have shown that improvements in spatiotemporal gait parameters do not necessarily translate into improvements in overall kinematic gait quality [61–64]. For example, in people with multiple sclerosis, 6 months of adapted physical activity improved stride length, gait speed, and cadence, whereas GPS and GVS remained largely unchanged, suggesting that changes in the overall kinematic gait pattern may require more targeted training [62]. In another rehabilitation context, a randomized cross-over trial in individuals with spastic cerebral palsy showed that traditional Thai massage reduced overall GPS by approximately 1.6° compared with standard physical therapy [63]. Although these studies differ from the present study in terms of population and intervention type, they provide indirect reference points for interpreting the magnitude of GPS change. Because minimal clinically important differences for GPS and GDI have not yet been established for prefrail older adults undergoing Tai Chi training, the present findings should be regarded as preliminary biomechanical evidence that Tai Chi may improve overall gait quality, and their clinical significance requires further validation.

The complementary group × time interaction analysis showed a generally similar pattern. After FDR correction, significant interactions were retained for gait speed, GPS, and GDI, suggesting that intervention-related longitudinal changes were more robust for walking speed and global gait quality than for most individual joint-specific kinematic variables. By contrast, although hip rotation deviation was significant in the baseline-adjusted post-intervention comparison, its group × time interaction was only nominally significant and did not remain significant after correction. This indicates that the evidence for differential longitudinal change in hip rotation was less stable than that for gait speed, GPS, and GDI. Overall, the present findings suggest that in prefrail older adults, the more robust gait-related effects of Tai Chi may be reflected primarily in gait speed and global gait quality indices. The positive findings for multiple individual joint-specific kinematic variables are better regarded as exploratory rather than confirmatory, and warrant further evaluation in studies with larger samples and adequate statistical power.

### Association between changes in postural stability and gait biomechanics

The correlation analyses in the present study should be regarded as exploratory. Their primary purpose was to provide an initial assessment of potential co-variation between changes in postural stability and changes in gait biomechanics after the intervention, rather than to test prespecified mechanistic hypotheses. To reduce analytical dimensionality while focusing on outcomes that appeared more responsive to the intervention, only representative variables showing nominal between-group differences in the ANCOVA analyses were included in the subsequent correlation analyses.

For the functional outcomes, the within-intervention-group analysis showed that ΔTUGT was negatively correlated with Δgait speed, whereas ΔSPPB was negatively correlated with Δankle ROM; in the full-sample sensitivity analysis adjusted for group, a nominal association in the same direction was also observed between ΔTUGT and Δgait speed. This pattern suggests that improvement in functional mobility may be related to improved walking efficiency [65], as the direction of increased gait speed and reduced TUGT completion time is clinically coherent. By contrast, the negative correlation between ΔSPPB and Δankle ROM suggests that improvement in overall functional performance did not necessarily occur in parallel with an increase in ankle range of motion. Because the SPPB reflects global functional status, and an increase in ankle ROM does not necessarily indicate functional improvement and may, in some cases, reflect compensatory adjustment, this finding should be interpreted with particular caution. Given that these associations did not remain significant after FDR correction, they are more appropriately viewed as exploratory directional signals.

For static balance, under the eyes-open condition, both Δpath length and Δsway velocity were negatively correlated with Δhip peak extension angle and positively correlated with Δhip ROM; the full-sample sensitivity analysis also identified nominal associations in the same direction involving hip peak extension angle. This pattern may suggest that changes in sagittal-plane hip kinematics are related to changes in postural control when visual information is available. More specifically, greater changes in peak hip extension were associated with greater reductions in COP path length and sway velocity. Previous studies have shown that reduced peak hip extension during walking in older adults is associated with poorer gait function and increased fall risk [66, 67]. Accordingly, the negative correlation between Δhip peak extension angle and COP sway measures may reflect coordinated changes between improved hip extension control during terminal stance and improved postural stability. By contrast, the positive associations between hip ROM and COP sway measures suggest that an increase in total hip excursion does not necessarily correspond to improved postural control, and may instead reflect greater movement variability or compensatory adjustment. Therefore, these findings are better interpreted as indicating an association between sagittal-plane hip movement patterns and postural regulation strategies, rather than implying a direct causal relationship. Under the eyes-closed condition, the within-intervention-group analysis showed that Δpath length and Δsway velocity were negatively correlated with Δstep length and Δhip peak extension angle, and positively correlated with Δhip ROM; in addition, Δsway ellipse area was negatively correlated with Δgait speed. In the full-sample sensitivity analysis, sway-related variables under eyes-closed conditions also showed several nominal associations in the same direction with ankle peak plantar flexion, ankle ROM, hip ROM, and hip peak extension angle. Compared with the eyes-open condition, the correlations observed under eyes-closed conditions involved a broader range of variables, including step length and both hip- and ankle-related measures, suggesting that when visual information is limited, changes in static postural control may be associated with a wider range of lower-limb gait kinematic characteristics [68–70]. In particular, the appearance of ankle-related variables may suggest that ankle propulsion and mobility characteristics contribute to postural regulation under conditions of reduced sensory input [71–73], as the role of the ankle in controlling anteroposterior sway and fine center-of-mass adjustments may become more prominent when visual compensation is reduced [71, 74]. However, because these associations did not remain significant after correction for multiple comparisons, and because correlation analyses cannot establish causality, the present findings can only be interpreted as suggesting potential gait–postural control relationships, rather than providing confirmatory evidence for the mechanisms by which Tai Chi improves static balance.

Overall, both the primary exploratory analysis within the intervention group and the group-adjusted full-sample sensitivity analysis identified several nominal associations that were biomechanically plausible in direction. However, none of these associations remained significant after FDR correction across the full correlation family. This pattern likely reflects, at least in part, the exploratory nature of the analyses, the use of change scores, the relatively limited sample size, and the statistical conservativeness introduced by the large number of tests. Therefore, the present correlation findings are better regarded as hypothesis-generating, highlighting potential biomechanical candidates for future investigation, rather than as confirmatory mechanistic evidence. Further studies with larger samples, more clearly prespecified mechanistic models, and more rigorous statistical control will be needed to determine the stability and reproducibility of these candidate associations.

### Clinical implications, limitations, and future directions

Prefrailty represents a critical and potentially reversible window of functional decline. The present study suggests that Tai Chi may improve functional mobility, balance-related performance, and static postural control in prefrail older adults, while also showing favorable effects on gait speed and global gait quality indices. These findings support the potential value of Tai Chi as a community-based and rehabilitation-oriented exercise strategy for this population.

Several limitations should be acknowledged. First, this was a single-center study with a relatively limited sample size, which may have reduced statistical power, particularly for individual joint-specific kinematic outcomes and exploratory correlation analyses. In addition, multiple outcome domains were assessed, and some positive findings did not remain significant after FDR correction. Therefore, secondary and exploratory outcomes, especially individual joint-specific kinematic variables and correlation findings, should be interpreted with caution. Second, the study did not include long-term follow-up or post-intervention reassessment of Fried Frailty Phenotype status. Although the baseline distribution of positive Fried components was reported, this study could not determine whether Tai Chi training promoted transition from prefrailty to robustness, nor whether the observed functional and biomechanical improvements were sustained over time. Third, several intervention-related factors may affect the generalizability of the findings. The Tai Chi intervention was delivered by a single experienced instructor, which helped maintain consistency in training content but may also have introduced instructor-specific effects. Future studies should include multiple trained instructors and, where feasible, balance or randomize instructors across groups or study sites. In addition, although supervised-session attendance was recorded and all analyzed participants met the prespecified adherence criterion, home practice time was not systematically recorded. Therefore, total Tai Chi practice dose and potential dose–response relationships could not be quantified. Finally, adverse-event monitoring was based mainly on researcher observation and participant reporting rather than a standardized adverse-event checklist or symptom diary. No serious or clinically significant adverse events attributable to Tai Chi were observed during the study period. However, minor transient exercise-related symptoms, such as mild muscle soreness, temporary aches, or fatigue, were not systematically quantified. Therefore, the safety findings should be interpreted as indicating the absence of serious or clinically significant intervention-related adverse events, rather than the complete absence of any minor exercise-related discomfort.

Despite these limitations, the present study provides preliminary randomized controlled evidence suggesting that a 12-week Tai Chi training program may improve functional mobility, static postural control, and overall gait quality in prefrail older adults. These findings support the potential value of Tai Chi as a safe, feasible, and multidimensional exercise intervention for this population. Nevertheless, larger, fully powered randomized controlled trials incorporating multicenter recruitment, multiple trained instructors, standardized adverse-event monitoring, systematic recording of supervised and home-based practice, repeated pre- and post-intervention frailty assessments, and long-term follow-up are needed to confirm these findings and clarify their long-term clinical relevance.

## Conclusion

This study provides preliminary randomized controlled evidence suggesting that a 12-week Tai Chi intervention may improve functional mobility, static postural control, and gait performance in prefrail older adults. Compared with the control group, participants in the Tai Chi group showed favorable improvements in functional assessments, including TUGT, BBS, and SPPB, together with reductions in COP sway-related parameters during static standing tasks. Three-dimensional gait analysis further suggested that Tai Chi training may improve gait speed and global gait-quality indices, including GPS and GDI, whereas findings for individual joint-specific kinematic variables should be interpreted cautiously as exploratory. Correlation analyses provided preliminary clues that changes in postural stability may be associated with alterations in selected gait biomechanical parameters; however, these findings should not be considered confirmatory mechanistic evidence. Overall, Tai Chi appears to be a safe, feasible, and multidimensional exercise intervention with potential benefits for balance and gait function in prefrail older adults. Larger, adequately powered studies with longer follow-up are needed to confirm these findings and clarify their clinical and mechanistic relevance.

## Data Availability

Data supporting the findings of this researsh are available from the corresponding author.

## Abbreviations

ANCOVA: Analysis of covariance
BBS: Berg balance scale
ChiCTR: Chinese clinical trial registry
COP: Center of pressure
GDI: Gait deviation index
GPS: Gait profile score
GVS: Gait variable score
IQR: Interquartile range
ROM: Range of motion
SD: Standard deviation
SPPB: Short physical performance battery
TUGT: Timed up-and-go test
χ²: Chi-square
MCID: Minimal clinically important difference
